# Supplementary material for: Machine Learning Accelerates Crystallization for Structure Determination
Source: Angew Chem Int Ed Engl. 2026 May 4;65(25):e1218503. doi: 10.1002/anie.1218503 (PMC13266958; doi:10.1002/anie.1218503)

checkCIF/PLATON report

Structure factors have been supplied for datablock(s) lcz081

THIS REPORT IS FOR GUIDANCE ONLY. IF USED AS PART OF A REVIEW PROCEDURE FOR PUBLICATION, IT SHOULD NOT REPLACE THE EXPERTISE OF AN EXPERIENCED CRYSTALLOGRAPHIC REFEREE.

No syntax errors found.   CIF dictionary   Interpreting this report

Datablock: lcz081

Bond precision:	C-C = 0.0232 A		Wavelength=1.54184
Cell:	a=13.2473(2)	b=22.9690(4)	c=42.2825(9)
	alpha=90	beta=90	gamma=90
Temperature:	100 K		

	Calculated	Reported
Volume	12865.6(4)	12865.6(4)
Space group	C 2 2 21	C 2 2 21
Hall group	C 2c 2	C 2c 2
Moiety formula	4(C15 H3 Ag3 F18 N6), 3(C10 H9 N) [+ solvent]	2(C15 H3 Ag3 F18 N6), 3(C5 H4.5 N0.5)
Sum formula	C90 H39 Ag12 F72 N27 [+ solvent]	C45 H19.50 Ag6 F36 N13.50
Mr	4160.92	2080.46
Dx,g cm-3	2.148	2.148
Z	4	8
Mu (mm-1)	15.764	15.764
F000	7920.0	7920.0
F000’	7959.17	
h,k,lmax	16,29,53	16,29,52
Nref	13854[7497]	13160
Tmin,Tmax	0.049,0.110	0.320,1.000
Tmin’	0.008	

```
Correction method= # Reported T Limits: Tmin=0.320 Tmax=1.000
AbsCorr = MULTI-SCAN

Data completeness= 1.76/0.95        Theta(max)= 78.556

R(reflections)= 0.0644( 12292)                 wR2(reflections)=
                                               0.1615( 13160)

S = 1.064                  Npar= 910
```

The following ALERTS were generated. Each ALERT has the format
test-name_ALERT_alert-type_alert-level.
Click on the hyperlinks for more details of the test.

Alert level B

```
PLAT342_ALERT_3_B Low Bond Precision on  C-C Bonds ..............     0.0232 Ang.
```

Author Response: Disordered structure.

Alert level C

```
PLAT213_ALERT_2_C Atom F01B           has ADP max/min Ratio .....        3.1 prolat
PLAT213_ALERT_2_C Atom N00I           has ADP max/min Ratio .....        3.1 oblate
PLAT213_ALERT_2_C Atom N00M           has ADP max/min Ratio .....        3.1 prolat
PLAT213_ALERT_2_C Atom F014           has ADP max/min Ratio .....        3.1 prolat
PLAT213_ALERT_2_C Atom F01O           has ADP max/min Ratio .....        3.1 prolat
PLAT213_ALERT_2_C Atom F023           has ADP max/min Ratio .....        3.7 prolat
PLAT234_ALERT_4_C Large Hirshfeld Difference F02V     --C02Y     .      0.21 Ang.
PLAT234_ALERT_4_C Large Hirshfeld Difference F030     --C02Y     .      0.24 Ang.
PLAT250_ALERT_2_C Large U3/U1 Ratio for <U(i,j)> Tensor(Resd    1)       2.3 Note
PLAT906_ALERT_3_C Large K Value in the Analysis of Variance ......     2.261 Check
PLAT911_ALERT_3_C Missing FCF Refl Between Thmin & STh/L=    0.600        10 Report
                9  1  0,   0 24  0,   0 24  1,   0 24  2,   0 24  3,   0 24  5,
                0 24  6,   0 24  8,   0 24 12,   0  0 50,
PLAT971_ALERT_2_C Check Calcd Resid. Dens.  1.10Ang From Ag01           1.90 eA-3
PLAT971_ALERT_2_C Check Calcd Resid. Dens.  1.11Ang From Ag03           1.89 eA-3
PLAT971_ALERT_2_C Check Calcd Resid. Dens.  1.44Ang From F02V           1.78 eA-3
PLAT971_ALERT_2_C Check Calcd Resid. Dens.  1.07Ang From Ag01           1.76 eA-3
PLAT971_ALERT_2_C Check Calcd Resid. Dens.  1.02Ang From Ag02           1.71 eA-3
PLAT971_ALERT_2_C Check Calcd Resid. Dens.  0.91Ang From Ag03           1.69 eA-3
PLAT971_ALERT_2_C Check Calcd Resid. Dens.  1.17Ang From Ag02           1.67 eA-3
PLAT971_ALERT_2_C Check Calcd Resid. Dens.  0.89Ang From Ag06           1.63 eA-3
PLAT971_ALERT_2_C Check Calcd Resid. Dens.  1.13Ang From Ag04           1.53 eA-3
PLAT977_ALERT_2_C Check Negative Difference Density on H01P      .     -0.38 eA-3
```

Alert level G

```
PLAT002_ALERT_2_G Number of Distance or Angle Restraints on AtSite          2 Note
PLAT003_ALERT_2_G Number of Uiso or U(i,j)  Restrained non-H-Atoms          9 Report
PLAT041_ALERT_1_G Calc. and Reported SumFormula    Strings  Differ     Please Check
              Calc: C90 H39 Ag12 F72 N27
```

```
             Rep.: C45 H19.50 Ag6 F36 N13.50
PLAT042_ALERT_1_G Calc. and Reported MoietyFormula Strings  Differ     Please Check
             Calc: 4(C15 H3 Ag3 F18 N6), 3(C10 H9 N)
             Rep.: 2(C15 H3 Ag3 F18 N6), 3(C5 H4.5 N0.5)
PLAT045_ALERT_1_G Calculated and Reported Z Differ by a Factor ...      0.500 Check
PLAT083_ALERT_2_G SHELXL Second Parameter in WGHT  Unusually Large     281.55 Why ?
PLAT164_ALERT_4_G Nr. of Refined C-H H-Atoms in Heavy-Atom Struct.          1 Note
PLAT172_ALERT_4_G The CIF-Embedded .res File Contains DFIX Records          1 Report
PLAT186_ALERT_4_G The CIF-Embedded .res File Contains ISOR Records          2 Report
PLAT242_ALERT_2_G Low    'MainMol' Ueq as Compared to Neighbors of       C01I Check
PLAT242_ALERT_2_G Low    'MainMol' Ueq as Compared to Neighbors of       C029 Check
PLAT242_ALERT_2_G Low    'MainMol' Ueq as Compared to Neighbors of       C02D Check
PLAT242_ALERT_2_G Low    'MainMol' Ueq as Compared to Neighbors of       C02I Check
PLAT242_ALERT_2_G Low    'MainMol' Ueq as Compared to Neighbors of       C02M Check
PLAT242_ALERT_2_G Low    'MainMol' Ueq as Compared to Neighbors of       C02O Check
PLAT242_ALERT_2_G Low    'MainMol' Ueq as Compared to Neighbors of       C021 Check
PLAT242_ALERT_2_G Low    'MainMol' Ueq as Compared to Neighbors of       C02J Check
PLAT242_ALERT_2_G Low    'MainMol' Ueq as Compared to Neighbors of       C02P Check
PLAT242_ALERT_2_G Low    'MainMol' Ueq as Compared to Neighbors of       C02U Check
PLAT242_ALERT_2_G Low    'MainMol' Ueq as Compared to Neighbors of       C02W Check
PLAT242_ALERT_2_G Low    'MainMol' Ueq as Compared to Neighbors of       C02Y Check
PLAT343_ALERT_2_G Unusual sp3      Angle Range in Main Residue for       C02Y Check
PLAT434_ALERT_2_G Short Inter HL..HL Contact F007     ..F01B     .       2.76 Ang.
                                              -1/2+x,1/2+y,z  =      5_455 Check
PLAT434_ALERT_2_G Short Inter HL..HL Contact F00O     ..F01O     .       2.84 Ang.
                                              1/2+x,-1/2+y,z  =      5_545 Check
PLAT605_ALERT_4_G Largest Solvent Accessible VOID in the Structure        233 A**3
PLAT720_ALERT_4_G Number of Unusual/Non-Standard Labels .........         122 Note
             Ag01    Ag02    Ag03    Ag04    Ag05    Ag06    F007    F008
             F009    F00A    F00B    F00C    N00D    F00E    N00F    F00G
             N00H    N00I    N00J    F00K    N00L    N00M    F00N    F00O
             F00P    F00Q    F00R    N00S    F00T    N00U    N00V    F00W
             F00X    F00Y    N00Z    N010    F011    N013    F014    N015
             F016    C017    F018    F019    C01A    H01A    F01B    C01C
             H01C    F01D    F01E    F01F    F01G    F01H    C01I    C01J
             H01J    C01K    C01L    H01L    F01M    C01N    F01O    C01P
             H01P    C01Q    C01R    H01R    C01S    C01T    F01U    C01V
             H01V    C01W    H01W    C01X    C01Y    H01Y    C01Z    C020
             C021    C022    H022    F023    C024    C025    H025    C026
             H026    C027    H027    C029    C02A    F02B    C02C    H02C
             C02D    C02E    C02F    H02F    C02H    H02H    C02I    C02J
             C02K    H02K    C02L    C02M    C02N    H02N    C02O    C02P
             C02Q    H02Q    C02R    H02R    C02T    C02U    F02V    C02W
             C02Y    F030
PLAT860_ALERT_3_G Number of Least-Squares Restraints ............          55 Note
PLAT868_ALERT_4_G ALERTS Due to the Use of _smtbx_masks Suppressed          ! Info
PLAT883_ALERT_1_G Absent Datum for _atom_sites_solution_primary ..     Please Do !
PLAT910_ALERT_3_G Missing FCF Reflection(s) Below Theta(Min)[Deg]=       3.85 Note
               0  0  2,
PLAT912_ALERT_4_G Missing # of FCF Reflections Above STh/L=  0.600        186 Note
PLAT933_ALERT_2_G Number of HKL-OMIT Records in Embedded .res File          8 Note
               0 24  0,   0 24  1,   0 24  2,   0 24  3,   0 24  5,   0 24  6,
               0 24  8,   0 24 12,
PLAT969_ALERT_5_G The 'Henn et al.' R-Factor-gap value ..........      2.828 Note
             Predicted wR2: Based on SigI**2  5.71 or SHELX Weight 15.19
PLAT978_ALERT_2_G Number C-C Bonds with Positive Residual Density.          0 Info
```

0 **ALERT level A** = Most likely a serious problem - resolve or explain
1 **ALERT level B** = A potentially serious problem, consider carefully
21 **ALERT level C** = Check. Ensure it is not caused by an omission or oversight
34 **ALERT level G** = General information/check it is not something unexpected

4 ALERT type 1 CIF construction/syntax error, inconsistent or missing data
37 ALERT type 2 Indicator that the structure model may be wrong or deficient
5 ALERT type 3 Indicator that the structure quality may be low
9 ALERT type 4 Improvement, methodology, query or suggestion
1 ALERT type 5 Informative message, check

Validation response form

Please find below a validation response form (VRF) that can be filled in and pasted into your CIF.

```
# start Validation Reply Form
_vrf_PLAT213_lcz081
;
PROBLEM: Atom F01B            has ADP max/min Ratio .....        3.1 prolat
RESPONSE: ...
;
_vrf_PLAT234_lcz081
;
PROBLEM: Large Hirshfeld Difference F02V     --C02Y     .       0.21 Ang.
RESPONSE: ...
;
_vrf_PLAT250_lcz081
;
PROBLEM: Large U3/U1 Ratio for <U(i,j)> Tensor(Resd    1)        2.3 Note
RESPONSE: ...
;
_vrf_PLAT906_lcz081
;
PROBLEM: Large K Value in the Analysis of Variance ......      2.261 Check
RESPONSE: ...
;
_vrf_PLAT911_lcz081
;
PROBLEM: Missing FCF Refl Between Thmin & STh/L=    0.600         10 Report
RESPONSE: ...
;
_vrf_PLAT971_lcz081
;
PROBLEM: Check Calcd Resid. Dens.  1.10Ang From Ag01            1.90 eA-3
RESPONSE: ...
;
_vrf_PLAT977_lcz081
;
PROBLEM: Check Negative Difference Density on H01P      .      -0.38 eA-3
RESPONSE: ...
;
# end Validation Reply Form
```

It is advisable to attempt to resolve as many as possible of the alerts in all categories. Often the minor alerts point to easily fixed oversights, errors and omissions in your CIF or refinement strategy, so attention to these fine details can be worthwhile. It is up to the individual to critically assess their own results and, if necessary, seek expert advice.

PLATON version of 17/09/2025; check.def file version of 17/09/2025

---

duplicate check

No duplication found

Datablock lcz081 - ellipsoid plot

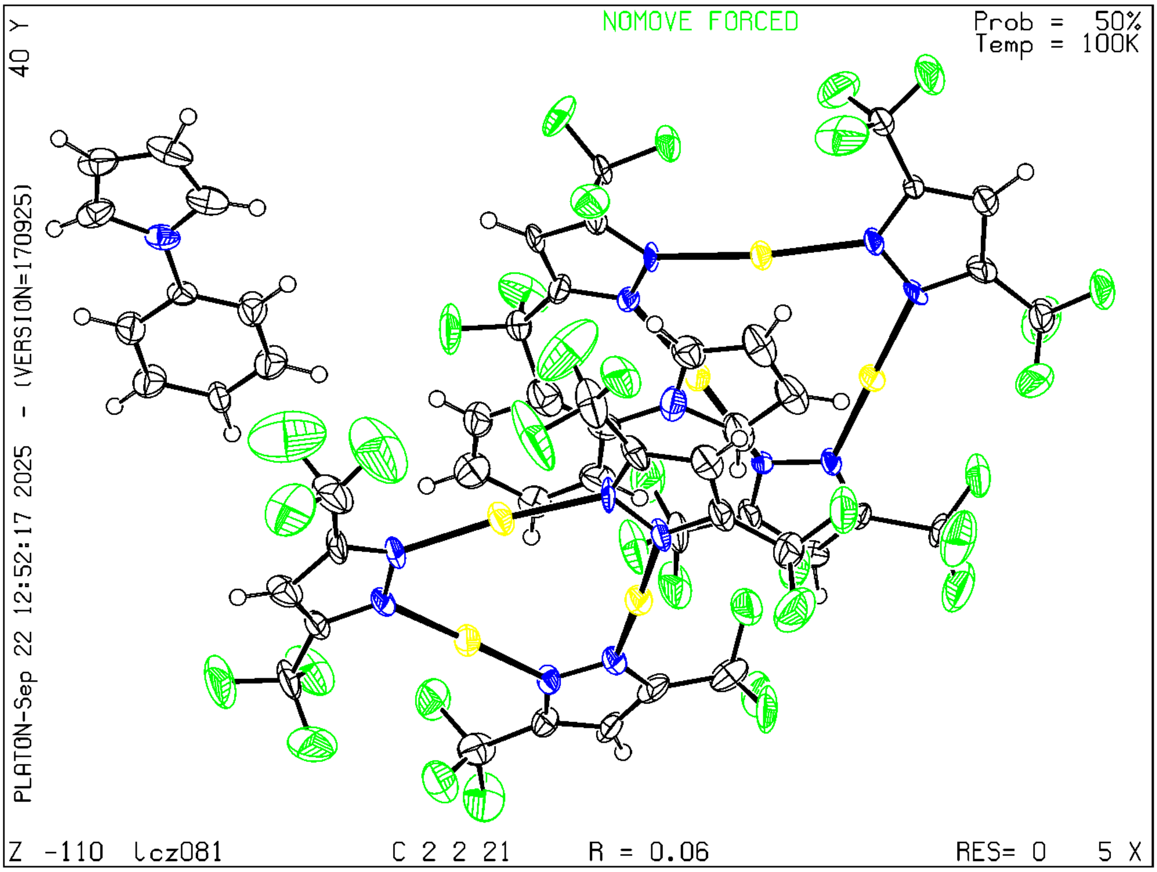

Supplement: Supplementary file 2 — Supporting File 2: anie72462‐sup‐0002‐DataFile.zip. [file ANIE-65-e1218503-s002.zip › supplementary_materials_crystallographic_data submit.zip!/cif_and_checkcif(1-114)/Ag3Pz3_81/Ag3Pz3_81_checkcif.pdf]
